# In memoriam: Gloria Petersen, PhD (1950-2023)

**DOI:** 10.1007/s10689-024-00406-y

**Published:** 2024-06-13

**Authors:** Hans Vasen

**Affiliations:** Department of Gastroenterology & Hepatology, Leiden, University Medical Center, Leiden, The Netherlands

**Keywords:** Research legacy, Pancreatic cancer research, Genetic epidemiology, Polyposis syndromes, Colorectal cancer, Cancer risk, Gene testing, Early detection, Johns Hopkins, Mayo Clinic

**Figure:**
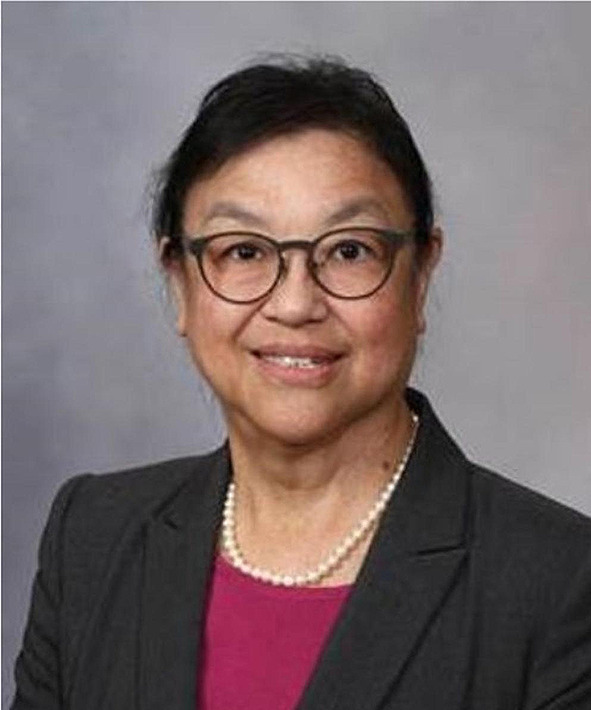
Gloria Petersen

On January 8, 2023, Gloria Petersen passed away at the age of 72. Gloria was a major contributor to the field of pancreatic cancer research. In this issue on hereditary pancreatic cancer, we want to highlight some of her major contributions to the field.

Petersen received her bachelor’s degree in 1972 from the University of California, Santa Barbara, her master’s degree in 1975 from the University of Oregon, and her doctoral degree in 1980 from the University of California, Los Angeles, all in physical anthropology. She completed a postdoctoral medical genetics fellowship at Harbor-UCLA Medical Centre in Torrance, California, in 1983. From 1983 to 1990, she served at Cedars-Sinai Medical Centre, Los Angeles, California. In 1990, she was offered a faculty position at Johns Hopkins in Baltimore, MD, where she conducted research in colorectal and pancreatic cancer. In 1999, she joined the Mayo Clinic Cancer Center as a professor in the Division of Epidemiology, Department of Quantitative Health Sciences. At Mayo, she primarily focused on pancreatic cancer, using the experience and skills developed in her earlier studies

Her research employed genetic epidemiology methods to understand the etiology of cancer, with an emphasis on hereditary forms of colorectal and pancreatic cancer. In the early 1990s, at Johns Hopkins, Petersen began researching familial adenomatous polyposis in collaboration with Francis Giardiello, Johan Offerhaus, Stanley Hamilton, Bert Vogelstein and others. She participated in many groundbreaking studies including those on the molecular diagnosis, phenotypic expression and chemoprevention of familial adenomatous polyposis. Her research also extended to other polyposis syndromes, including studies on cancer risk in Peutz-Jeghers syndrome and the discovery of involvement of *BMPR1A* in the genesis of Juvenile Polyposis. Additionally, she made contributions to the field of Lynch syndrome, conducting studies on microsatellite instability (MSI) in young patients with colorectal cancer, studies comparing MSI with immunohistochemistry testing of colorectal tumors, and researching familial colorectal cancer type X in collaboration with Noralane Lindor. Following discovery of the genetic basis for various hereditary CRC syndromes, she played a leading role in establishing guidelines for gene testing and counseling.

In the mid-1990s, Petersen began researching in pancreatic cancer in collaboration with Ralph Hruban, Michael Goggins, Alison Klein, Stijn Tersmette and Mimi Canto at Johns Hopkins. She contributed to numerous significant studies, including estimating prospective risk of pancreatic cancer in families according to the number of affected relatives, outcomes of surveillance, the association between diabetes and pancreatic cancer, genetic and non-genetic risk factors for pancreatic cancer, genetic alterations during the progression from precursor lesion to pancreatic cancer, and the prevalence of pathogenic germline variants in patients with sporadic and familial pancreatic cancer.

After joining the Mayo Clinic, Petersen and others established the Pancreatic Cancer Genetic Epidemiology (PACGENE) consortium in 2002, aiming to identify susceptibility genes in familial pancreatic cancer. She also initiated and/or participated in many other national and international consortia such as the Pancreatic Cancer Case-Control Consortium (PanC4), the Pancreatic Cancer Cohort Consortium (PanScan), and the Pan-Cancer Analysis of Whole Genomes Consortium (PCAWG). These collaborations led to numerous groundbreaking studies.

Apart from her countless research contributions, Gloria Petersen is remembered as a fantastic mentor, investigator, and friend, as stated by Allison Rosenzweig PhD in her obituary for Petersen. Her writing included reactions from other colleagues to Petersen’s passing: “Gloria has been a guidepost in my life for 25 years”, shared Alison Klein PhD, a former trainee and longstanding collaborator, “Her work will continue through the collaborative networks she helped to establish as well through her mentees, collaborators, and friends.” “Gloria will keep inspiring us to do better”, according to Suresh Chari MD, former colleague at Mayo Clinic. “I could walk into Gloria’s office any time, unannounced, and she had always time to discuss yet another crazy idea”. He added, “Keywords that come to my mind when I think of Gloria: unflappable, selfless, generous, dedicated, passionate, data driven, scientific rigor, open to outside-the-box ideas”. “Gloria has been such an amazing and steadfast champion for Pancreatic Cancer Action Network (PanCAN)”, said Julie Fleshman, chairman of PanCAN, ”I am incredibly grateful for her uplifting spirit, motivating leadership, and the critical advice she has given me and the organization over the years.”

Through all her research contributions, Petersen aimed to reduce the devastation of this silent cancer on patients and families. Her husband, Wes Petersen, recalled her saying a few years ago “I want to make pancreatic cancer a survivable disease through early detection and the use of targeted gene therapies.” By the time of her death, part of her vision had been realized, as an increasing number of patients were surviving their cancer due to early detection. Her pioneering work in pancreatic cancer research has profoundly impacted the field and will continue to inspire and guide future advancements. Her dedication, kindness, and scientific brilliance have left an enduring legacy.

## Data Availability

No datasets were generated or analysed during the current study.

